# Placing the leading limb closer to an obstacle reduces collision of the trailing limb: an investigation in a virtual environment

**DOI:** 10.3389/fspor.2024.1411037

**Published:** 2024-08-14

**Authors:** Tomoki Hakamata, Juntaro Sakazaki, Takahiro Higuchi

**Affiliations:** ^1^ Department of Health Promotion Science, Tokyo Metropolitan University, Tokyo, Japan; ^2^ Department of Rehabilitation, Kasai Central Hospital, Tokyo, Japan; ^3^ Division of Physical Therapy, Department of Rehabilitation Sciences, Faculty of Health Care Sciences, Chiba Prefectural University of Health Sciences, Chiba, Japan

**Keywords:** walking, obstacle crossing, collision avoidance, virtual reality, motion analysis

## Abstract

**Introduction:**

When walking and stepping over an obstacle of a certain height, tripping occurs more frequently with the trailing limb than the leading limb. The present study was designed to address whether collisions involving the trailing limb can be improved with experimental manipulation of the placement of the leading limb after stepping over an obstacle. We used an immersive, virtual obstacle-crossing task to ensure that the collision was not improved simply due to the experience of physical collision with an obstacle.

**Methods:**

Fourteen young participants (12 males and 2 females, 28.7 ± 3.5 years) were required to walk and step over a virtual horizontal pole under one of four conditions. In three conditions, participants were required to place their leading foot on a square target located along their walking path after crossing the obstacle. The target was positioned so that it was relatively close to the obstacle (10 cm from the obstacle, referred to hereafter as the closer condition), at a position that would naturally be stepped on in successful trials without a collision (20 cm from the obstacle, the middle condition), or relatively far from the obstacle (40 cm from the obstacle, the farther condition). For the fourth condition, participants were free to select where they would step after stepping over the obstacle (the control condition).

**Results and discussion:**

The results showed that the collision rate of the trailing limb was significantly lower under the closer condition than under the other three conditions. Compared to the control condition, under the closer condition the movement of the trailing limb was modified so that obstacle crossing was performed at approximately the moment when the height of the toe of the trailing limb was higher, and the walking speed was slower. These findings suggest that placing the foot of the leading limb closer to the obstacle after crossing the obstacle may ensure safe obstacle avoidance by the trailing limb.

## Introduction

Obstacle crossing during walking is essential for preventing trip-induced falls and injuries. Previous studies have shown that tripping occurs more frequently with the trailing limb than with the leading limb (1–4). Several reasons exist for collisions being more frequent with the trailing limb; compared to the leading limb, the foot of the trailing limb is often placed closer to an obstacle before stepping over it (1, 2, 5, 6), it has a lower clearance height (7), and it moves faster at the moment of stepping over the obstacle (7). Moreover, collisions involving the trailing limb are considered to be more likely given that it is out of sight at the moment of stepping over an obstacle (2). Previous studies have suggested that the trailing limb may have a lower priority for cognitive information processing when planning movement (8), possibly because the leading limb has a higher risk of causing falling when it collides with an obstacle compared to the trailing limb (1). Therefore, it is helpful to consider how to control the trailing limb in order to ensure that collisions are avoided and how to lead people who have difficulties in avoiding collisions, such as older adults (9), in order to improve their behavior.

Even if the movement of the trailing limb could potentially be altered by direct intervention in the movement, such an approach may not be ideal if it is dependent upon prioritizing the cognitive information processing required for planning the movement of the trailing limb over that required for the leading limb. In fact such a change could increase the risk of falling or tripping with the leading limb (1). We therefore considered that intervention in the landing position of the leading limb after stepping over an obstacle could improve control of the trailing limb. In particular, we considered that placing the foot of the leading limb relatively close to an obstacle after stepping over an obstacle might be effective for avoiding tripping with the trailing limb. This is because it could lead to (a) slower walking speed due to more careful control of the leading limb during obstacle crossing in order to avoid collision with a closer obstacle, (b) the foot placement of the trailing limb being not too close to an obstacle, which could help avoid tripping during the initial phase of obstacle crossing, and (c) higher foot clearance as a result of obstacle crossing during the latter phase of crossing. Moreover, recent studies suggest that there is interaction between the left and right, or leading and trailing, limbs in the motor control system (10, 11). If so, precise control of the leading limb could facilitate more precise control of the trailing limb while avoiding the need to prioritize planning the movement of the trailing limb during cognitive information processing.

The aim of the present study was to address whether collisions involving the trailing limb could be minimized with experimental manipulation of the placement of the leading limb after stepping over an obstacle. We hypothesized that manipulating foot placement of the leading limb closer to the obstacle after obstacle crossing would be effective for avoiding collisions of the trailing limb. We tested this hypothesis with younger adults to determine whether is worth implementing in the future with older adults. We also tested this hypothesis in a virtual reality (VR) environment in an effort to create a situation in which no physical collision with an obstacle occurred. This was necessary because collisions act as powerful feedback, indicating that the behavior was unsuccessful, and increase motivation to change behavior, even if experimental manipulation under each condition is not effective.

## Materials and methods

### Participants

Fourteen young individuals (12 males and 2 females, mean age = 28.7 years, SD = 3.5 years) participated in the experiment. The sample sizes were determined based on similar studies (12, 13) and an *a priori* power analysis assuming repeated measures analysis of variance. We calculated the sample size based on the power analysis performed with the G∗ Power software package (14) using the following parameters: effect size = 0.5, signifiance threshold (*α*) = 0.05 and power levels (1-β) = 0.8. The effect size of 0.5 was employed based on a previous report (12). All participants had normal or corrected-to-normal vision. Their mean standing height was 171.8 cm (SD = 9.2 cm) and their limb length was 85.5 cm (SD = 4.9). The right limb was dominant in all participants. Testing was approved by the Ethics Committee of Tokyo Metropolitan University, Japan (H5-25). Written informed consent was obtained from all participants in accordance with the Ethics Committee of Tokyo Metropolitan University and the Declaration of Helsinki. Participants received a bookstore gift card as a reward for their participation.

### Apparatus

**Figure 1:**
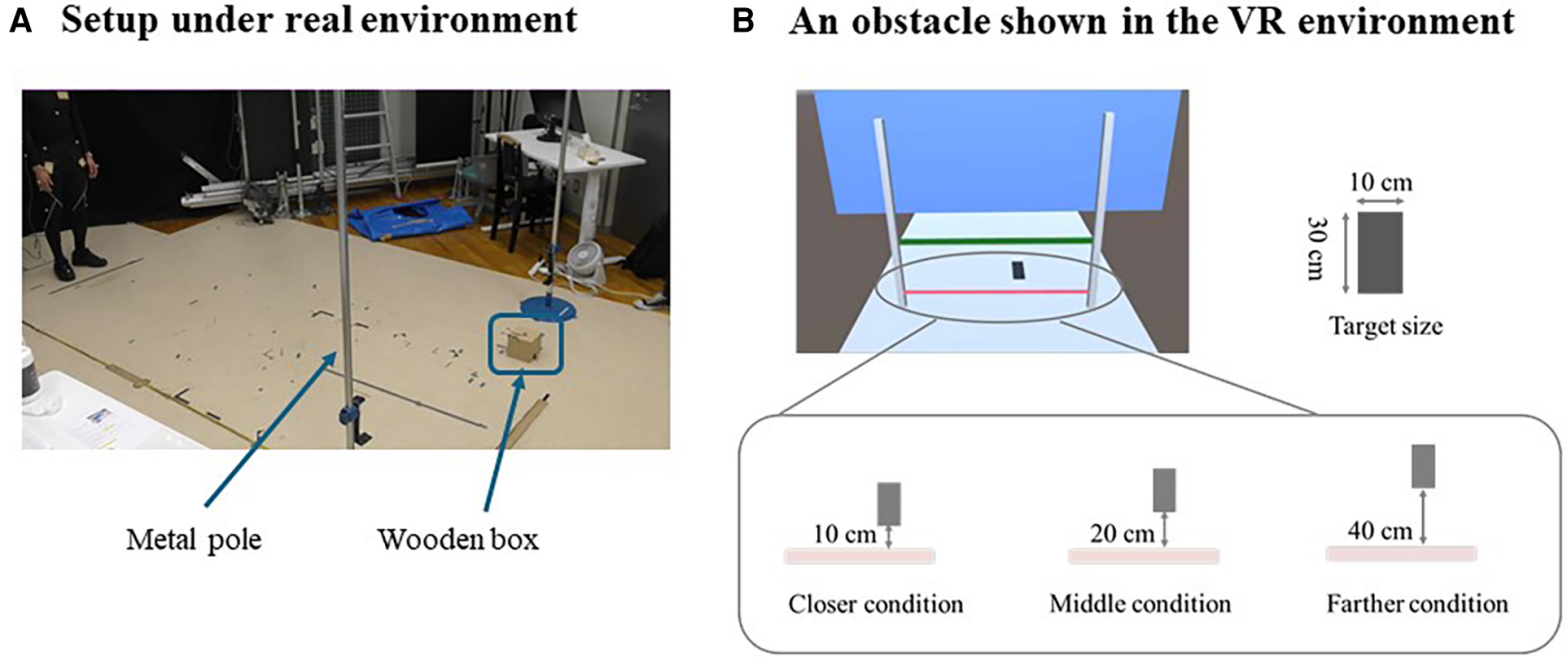
(**A**) experimental setup in a real-world environment. A wooden box was placed outside the walking path and was used to determine the position where the virtual object would be displayed in a virtual environment. Two vertical metal Poles were also used to indicate the obstacle height using the location of the reflective markers on the pole. (**B**) (Top left) an obstacle (horizontal pole) shown in a virtual environment; (Top right) A black square presented as a target on the ground; (Bottom) Three out of the four experimental conditions in which a target was presented.

The experiment was conducted in a room measuring 6.7 m × 4.9 m (Figure 1A). Participants were asked to walk for 4 m from a starting line on a walking path measuring 5.5 m long × 1.25 m wide. A desktop computer (OMEN by HP Obelisk Desktop 875-1xxx, HP, USA) was used for data collection and stimulus presentation. Participants wore a head-mounted display (HMD) (Oculus Rift S, USA) with a resolution of 1,280 pixels × 1,440 pixels per eye and a diagonal viewing angle of 111 degrees. The HMD was wired (the length of the cable was 5 m) to ensure stable communication. To reduce the feeling of being pulled by the cable while walking, the cable was suspended from a circular sling attached to the ceiling. The spatial positions of the HMD, the entire body, and obstacles were captured by 18 cameras (OQUS and MIQUS, Qualisys, Sweden) for three-dimensional motion analysis at a sampling frequency of 60 Hz. The cameras tracked a total of 50 markers to measure the location of the HMD, the wooden box used to capture the location of a virtual obstacle, poles, start and stop positions, and the participant's whole body (see Appendix A for details). Three-dimensional marker positions were streamed from the software for the motion analysis (Qualisys Track Manager) to the Unity game engine (Unity Technologies, USA) with a delay of approximately 40 ms. Visual 3D version 6 (C-Motion) was used for data processing.

A wooden box (Figure 1A) was used to represent the location of a virtual object (a long horizontal bar) that was presented in the virtual environment and to facilitate capture of the location of the virtual object for three-dimensional motion analysis (i.e., since the virtual objects cannot be captured, we captured the wooden box which represented the location of the virtual bar). A total of five reflective markers were placed on the box in a noncollinear arrangement to represent the box as a rigid body. Its three-dimensional position was streamed to the Unity game engine to display the virtual bar and was used for conducting the subsequent motion analysis to test our hypothesis. The wooden box was placed outside the walking path so that physical collisions with the obstacle did not occur. Two vertical metal poles, one on each side of the walking path, were used to visually confirm the location of the obstacle, as well as to indicate the obstacle height using the location of a marker on the pole as a reference point during motion analyses.

The VR environment was set up as shown in Figure 1B. The walking path was 5.5 m long × 1.25 m wide and the two vertical metal poles indicated the location of the obstacle in the real environment. A virtual obstacle, a long horizontal pole to step over, was presented in pink and was positioned 3 m from the starting line. The height of the pole was 20% of the participant's lower-limb length, represented as the length from the greater trochanter to the plantar surface. The diameter of the horizontal pole was 2 cm. The height of the obstacle used in this study has been employed in previous studies (15–17). In a preliminary study, we confirmed that trailing limb collisions typically occurred at a height equivalent to 20% that of the lower-limb length (see Appendix B for details). The preliminary study was necessary to examine the effect of the position of the stepping mark on the collision rate. A target, consisting of a black square (30 cm long × 10 cm wide), was placed ahead of the obstacle. A green line positioned 1 m beyond the obstacle, marked the position to stop, while a red tape, located 3 m in front of the obstacle, marked the starting position for walking. Two virtual blue walls were located at each end of the walking path to ensure that participants stopped walking and did not collide with the physical walls of the room. Other than the walking path, which was colored light blue, the floor was colored gray and the surrounding area was colored light blue. These are the default colors in the Unity program. No other objects were presented in the virtual environment.

### Task and procedure

The experimental task was to walk in the virtual environment and step over a virtual obstacle (a long horizontal bar) located 3 m from the walking starting position. For each trial, participants wearing an HMD stood in front of the starting position, indicated by a red line in the VR environment. After receiving a verbal instruction to start walking walk by the experimenter, participants started walking at a comfortable speed. Initially, they were asked to step over the virtual horizontal pole with their right limb while trying to avoid colliding with it. They selected which limb to use when initiating walking so as to comfortably step over the pole with their right limb. They stopped walking when they reached the green line on the walking path. We asked them to close their eyes while waiting on the green line. This was necessary to prevent VR sickness, caused by the image distortion that occurs at the end of the motion analysis in each trial. Participants opened their eyes and returned to the starting position after being requested to do so by the experimenter. No visual information about body movement was collected.

The task was performed under four experimental conditions: closer, middle, farther, and control (see Figure 1B). For three of these conditions, participants were requested to place the foot of their leading limb within a designated area (a black target) on the walkway after stepping over the obstacle. The target was located at a position that would naturally be stepped upon for successful trials without a collision (referred to as the middle condition), at a point 10 cm closer to the obstacle than the position used for the middle condition (the closer condition), or at a point 20 cm farther from the obstacle as compared to the position of the middle condition (the farther condition). For the fourth condition, no mark was placed after the obstacle, and participants were asked to step freely over the obstacle (control condition). A previous study showed that to successfully cross an obstacle in a real environment, the horizontal distance between the obstacle and the leading limb just after stepping over an obstacle averaged 30 cm (18). However, this distance may not be the same in a VR environment, given that the perception of distance differs between VR and real environments (19, 20). Therefore, we conducted a preliminary experiment to measure the average horizontal distance between the obstacle and the leading limb for successfully crossing the obstacle in our VR environment. The results showed that the average distance was 20 cm (see Appendix B for details). Based on these findings, we set the distance between the target and the obstacle.

Participants performed 10 trials for each experimental condition to give a total of 40 main trials. Prior to performing the main trials, three practice trials were conducted for each condition to allow participants to familiarize themselves with the experimental procedure. This was necessary because none of the participants were familiar with walking in a VR environment. For all conditions, except the control condition, the participants were also requested to step onto the target with their leading limb. Because this additional request also required familiarization, we decided to start the experiment with the control condition for all participants. The order of the other three conditions was randomized.

### Data analysis

**Figure 2:**
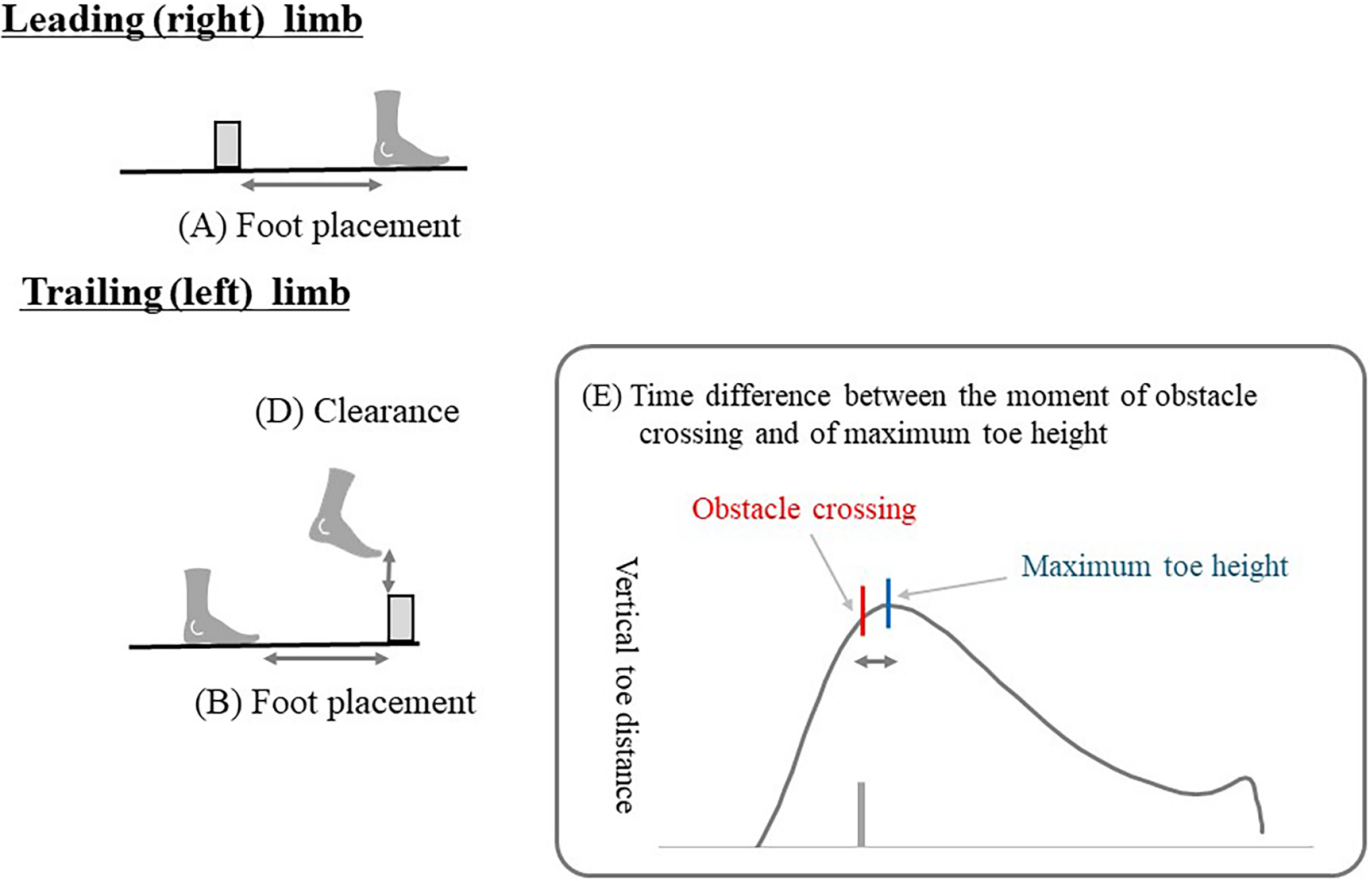
Dependent variables used to show the spatial or temporal characteristics of stepping over an obstacle. (**A**) foot placement of the leading limb after stepping over the obstacle, (**B**) foot placement of the trailing limb before the obstacle, (**D**) clearance height of the trailing limb, and (**E**) time difference between the moment of obstacle crossing and the maximum toe height of the trailing limb.

The following seven variables were measured to assess how participants stepped over the obstacle (Figure 2): (A) foot placement of the leading limb after stepping over the obstacle, (B) foot placement of the trailing limb before the obstacle, (C) trailing limb collision rate, (D) clearance height of the trailing limb, (E) time difference between the moment of obstacle crossing and the maximum toe height of the trailing limb, (F) walking speed at the time of obstacle crossing with the trailing limb, and (G) step length when crossing an obstacle. Since no collisions were observed involving the leading limb, we did not include the data in our statistical analysis.

The placement of the foot of the leading limb after the obstacle was defined as the horizontal distance between the heel marker of the leading limb and the obstacle at the moment of the initial step after stepping over the obstacle. This parameter was measured to address whether the experimental manipulation under the closer, middle, and farther conditions was appropriate (i.e., 10 cm, 20 cm, and 40 cm, respectively). The foot placement of the trailing limb before the obstacle at the moment of obstacle crossing with the leading limb was defined as the horizontal distance between the second metatarsal marker of the trailing limb and the obstacle before stepping over the obstacle. The collision rate of the trailing limb was determined based on the vertical distance between the second metatarsal marker of the trailing limb and the marker on the vertical pole, which represented the height of the vertical obstacle. A collision was determined to have occurred when the distance value was below zero. The clearance height of the trailing limb was defined as the vertical distance between the second metatarsal marker of the trailing limb and the obstacle at the moment of stepping over the obstacle with the trailing limb. The time difference between the moment of obstacle crossing and of maximum toe height of the trailing limb was the difference between the time when stepping over with the trailing limb and when the toe of the trailing limb reached the maximum height. If the difference was zero, then it follows that the obstacle was stepped over at the moment that the trailing limb was raised to the highest point. Positive values of difference indicate that the trailing limb was raised to its highest point after the moment of obstacle crossing. Walking speed at the time of obstacle crossing with the trailing limb was calculated as the AP direction COM velocity at the moment the trailing limb crossed the obstacle marker. Step length was defined as the horizontal distance between the heel markers of the leading and trailing limbs at the time the leading limb landed. Prior to calculations, all three- dimensional data were processed with a Butterworth filter using a cutoff frequency of 4 Hz.

For statistical analyses, we initially conducted a one-way MANOVA (foot placement) with repeated measures for the following set of dependent variables: collision rate, walking speed, trail foot placement, clearance, and time difference. A MANOVA was conducted to explore potential interactions among variables. We then conducted a one-way repeated analysis of variance (ANOVA within-factor) to perform detailed analyses on the individual dependent variables. Since the distribution for the collision rate of the trailing limb was not normal, the data were adjusted using an arcsine transformation for statistical analysis by one-way repeated ANOVA. When a significant main effect was identified, Scheffe's post-hoc tests were carried out to estimate the significant differences. The level of significance for all analyses was set at *p* < 0.05. Notably, all of the participants started the task from the control condition. To confirm that this did not produce severe order effects, particularly on the collision rate, we sorted the data for the collision rate obtained from the four experimental conditions in the order in which they were measured, regardless of the condition of foot placement, and statistically analyzed the data using a one-way (order) ANOVA.

## Results

The results of the MANOVA showed a main effect of foot placement [Wilks’ λ = 0.51, F (15,132.9) = 2.41, *p* = 0.004, $\eta_{P}^{2}=0.19$], suggesting that experimental manipulation of the leading foot placement significantly impacts the outcomes, despite the interdependence of the dependent variables.

The ANOVA results are as follows: the mean foot placement of the leading limb after stepping over the virtual obstacle under each experimental condition is shown in Table 1. A main effect was significant [F (3, 39) = 101.29, *p* < 0.001, $\eta_{G}^{2}=0.72$]. The foot placement of the leading limb after stepping over the virtual obstacle was significantly shorter under the closer condition compared to the middle (*p* < 0.001), control (*p* < 0.001), and farther conditions (*p* < 0.001). Conversely, the foot placement was significantly longer under the farther condition than under the closer (*p* < 0.001), control (*p* < 0.001), and middle conditions (*p* < 0.001). In each condition where a target was presented, it was confirmed that the leading limb successfully landed on the target (e.g., 20.67 cm in the middle condition where the target was placed at 20 cm). Similar results were obtained as in the preliminary study (see Appendix B for details).

**Table 1:** Mean and standard deviation of five dependent variables under four experimental conditions.

Conditions	Closer^b^	Middle^b^	Farther^b^	Control^b^	Statistics
Foot placement of the leading limb after stepping over the obstacle (A)^a^	12.88 (4.88)	20.67 (4.58)	40.56 (8.91)	23.15 (6.46)	Cl < M, F, Co F > Cl, M, Co
Foot placement of the trailing limb before the obstacle (B)^a^	23.78 (7.98)	22.92 (7.66)	18.84 (6.24)	26.18 (7.18)	F < Cl, M, Co M < Co
Clearance height of the trailing limb (D)^a^	13.33 (9.38)	9.75 (11.18)	6.41 (9.87)	11.97 (10.83)	Cl > M, F Co > F
Walking speed at the time of obstacle crossing with the trailing limb (F)^a^	0.79 (0.15)	0.86 (0.15)	0.98 (0.12)	0.89 (0.13)	Cl < M, F, Co F > Cl, M, Co
Step length when crossing an obstacle (G)^a^	52.42 (8.66)	59.7 (9.31)	76.18 (6.9)	66.57 (8.3)	Cl < M, F, Co F > Cl, M, Co M < Co

Cl, closer; M, middle; F, farther; Co, control.

^a^ Uppercase letters in parentheses correspond to the letter for dependent variables.

^b^ Standard deviation in parentheses.

The mean trail foot placement before the obstacle under each experimental condition is shown in Table 1. A main effect was significant [F (3, 39) = 11.47, *p* < 0.001, $\eta_{G}^{2}=0.12$]. The foot placement was significantly closer under the middle condition than that under the control condition (*p* = 0.049). The foot placement was also significantly closer under the farther condition than under the closer (*p* = 0.003), control (*p* < 0.001), and middle conditions (*p* = 0.014). Regarding the additional analysis to determine if an order effect influenced the collision rate results, the one-way ANOVA revealed no significant main effect of order [F (1.9, 24.72) = 0.77, *p* = 0.466, $\eta_{G}^{2}=0.01$], suggesting that order effects were negligible.

**Figure 3:**
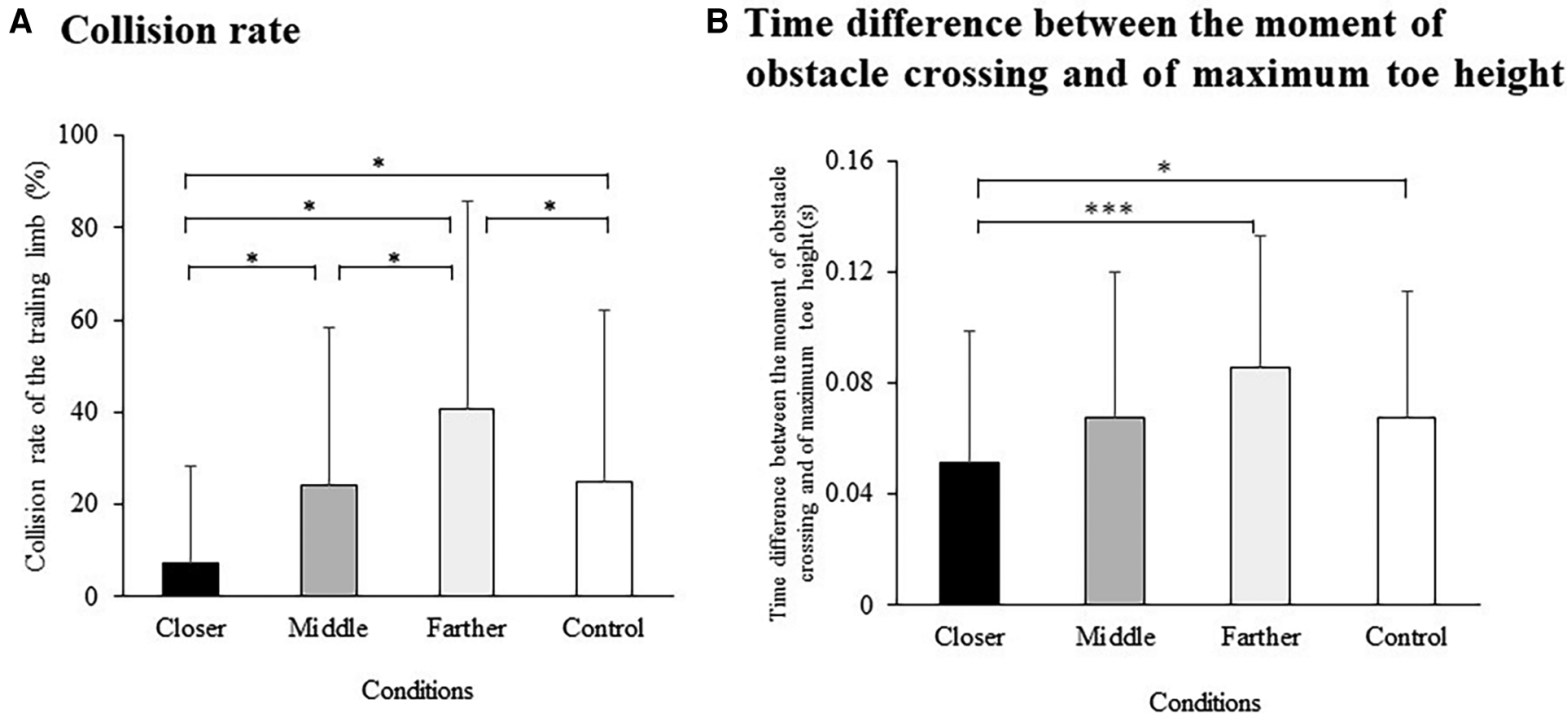
(**A**) mean and standard deviation of the collision rate involving the trailing limb under four experimental conditions; and (**B**) time difference between the moment of obstacle crossing and of maximum toe height under four experimental conditions. Significance levels are indicated by *p < 0.05 and ***p < 0.001, as determined by Scheffe's *post-hoc* test.

The mean collision rate of the trailing limb under each experimental condition is shown in Figure 3A. An ANOVA applied to the data adjusted using the arcsine transformation revealed a significant main effect [F (1.68, 21.88) = 9.06, *p* = 0.002, $\eta_{G}^{2}=0.11$]. Specifically, the collision rate was significantly lower under the closer condition compared to the middle (*p* = 0.023), control (*p* = 0.039), and farther conditions (*p* = 0.023). Conversely, the collision rate was significantly higher under the farther condition than under the closer (*p* = 0.023), control (*p* = 0.039), and middle conditions (*p* = 0.039).

The mean clearance height of the trailing limb under each experimental condition is shown in Table 1. A significant main effect was observed [F (1.93, 25.03) = 8.98, *p* = 0.001, $\eta_{G}^{2}=0.06$], with the clearance height significantly higher under the closer condition compared to the middle (*p* = 0.002) and farther conditions (*p* < 0.001). Additionally, the clearance height was lower under the farther condition than under the control condition (*p* = 0.028).

The mean time difference between the moment the obstacle is crossed and the maximum toe height of the trailing limb is shown in Figure 3B. The ANOVA showed a significant main effect [F (2.16, 28.06) = 5.79, *p* = 0.006, $\eta_{G}^{2}=0.06$]. This difference was significantly smaller under the closer condition compared to the control (*p* = 0.047) and farther conditions (*p* < 0.001).

The mean walking speed at the moment of crossing the obstacle with the trailing limb under each experimental condition is shown in Table 1. A significant main effect was observed [F (2.08, 27.02) = 27.59, *p* < 0.001, $\eta_{G}^{2}=0.2$]. Walking speed was significantly slower under the closer condition compared to the middle (*p* < 0.001), control (*p* < 0.001), and farther conditions (*p* < 0.001). Walking speed was significantly faster under the farther condition compared to the closer (*p* < 0.001), control (*p* = 0.003), and middle conditions (*p* < 0.001).

The mean step length when crossing an obstacle under each experimental condition is shown in Table 1. A significant main effect was observed [F (3, 39) = 55.21, *p* < 0.001, $\eta_{G}^{2}=0.52$]. Step length was significantly shorter under the closer condition compared to the middle (*p* < 0.001), control (*p* < 0.001), and farther conditions (*p* < 0.001). Step length was significantly longer under the farther condition compared to the closer (*p* < 0.001), control (*p* < 0.001), and middle conditions (*p* < 0.001). Step length was also shorter under the middle condition than under the control condition (*p* = 0.009).

## Discussion

In this study, we examined whether experimental manipulation involving shortening of the horizontal distance between the obstacle and leading limb immediately after stepping over an obstacle would be effective for avoiding collisions of the trailing limb. The results supported the hypothesis in that the collision rate of the trailing limb was significantly lower under the closer condition than other conditions (Figure 3A). We hypothesized that experimental manipulation of the closer condition would reduce the collision rate of the trailing limb due to slower walking speed and more careful control of the leading limb, foot placement of the trailing limb not being too close to an obstacle, and higher foot clearance. Among these expectations, the present findings showed that a slower walking speed and foot placement of the trailing limb not being too close to an obstacle supported our hypothesis. Although the clearance height of the trailing limb tended to be larger in the closer condition (Table 1), this increase was not significant. Placing the leading limb closer to the obstacle effectively maintains a reasonable distance between the trailing limb and the obstacle. However, this placement also increased the risk of collisions with the trailing limb. Consequently, the leading limb may have been more carefully controlled, which resulted in a slower walking speed. Importantly, the step length was significantly shorter under the closer conditions compared to the other conditions (Table 1). This reduction in stride length contributed to the slower walking speed observed in the closer condition.

Trade-offs between speed and accuracy is a robust phenomenon in human motor performance (21, 22). Previous studies have shown that trade-offs occur in obstacle avoidance (23), as well as walking and stepping tasks (24). Previous studies have shown that decreasing walking speed is a strategy employed to increase stability in passing (25, 26). Thus, experimental manipulations that shorten the horizontal distance between the obstacle and the leading limb immediately after stepping over an obstacle could improve the accuracy of trailing limb movement. This improvement may be facilitated by strategies such as shortening the stride length or decreasing the walking speed, highlighting a trade-off between speed and accuracy.

An unexpected but interesting finding was that the time difference between the moment of obstacle crossing and the maximum toe height of the trailing limb decreased significantly under the closer condition (Figure 3B; see also Figure 2 for the meaning of the results). This suggests that, although there was no increase in the maximum toe height, the risk of collision with the trailing limb was reduced because participants crossed the obstacle when the trailing limb was at its maximum height. Based on these findings, we propose that the experimental manipulation of the leading limb placement after stepping over an obstacle resulted in temporal rather than spatial adjustments of the trailing limb trajectories.

Placing the leading limb closer to an obstacle has generally been considered to increase collision risk (27). To the best of our knowledge, no studies have specifically examined the effects of placing the leading limb closer to an obstacle. However, considering that vision is crucial for controlling the placement of the leading limb (4, 28), we considered that avoiding a collision with an obstacle is possible through careful vision monitoring, even when the leading limb is positioned close to the obstacle. Additionally, if there is an interaction between the left and right, or leading and trailing, limbs in the motor control system (10, 11), then precise control of the leading limb would influence the subsequent control of the trailing limb. Some researchers have also suggested that the trailing limb may be deprioritized in cognitive information processing during movement planning (8). For this reason, there is concern that direct manipulation of the trailing limb may not preserve its priority and collisions involving the leading limb may increase. Therefore, our aim was to safely avoid collisions involving the trailing limb while preserving the prioritization of cognitive processing by specifically manipulating the placement of the leading limb. The results of the present study show that placing the leading limb closer to the obstacle, such as approximately 10 cm away, effectively reduced the number of collisions involving the trailing limb.

This study has several limitations. First, only the immediate effect of manipulating the placement of the foot on the leading limb was examined. It may be necessary to investigate retention effects, as in a previous study (26), and also to examine the effects of long-term duration. Second, the extent to which the task, involving manipulation of the placement of the leading limb closer to the obstacle, can be generalized to real-world environments remains unknown. Collision generally occurs more frequently in the VR environment—25% under the control conditions in this study and 26% in a previous study (18)—as opposed to 0.6% in the real environment (1). Future studies are needed to test whether the experimental manipulation conducted in the VR environment of the present study would also be effective in the real-world environment. Third, collision was determined based on the vertical distance between the second metatarsal and the obstacle. Practically, even though there is a space between the two, collision would occur if individuals were wearing thick-soled shoes. This suggests that collision avoidance in practical settings involves adaptation to the constraints imposed by footwear. Future studies need to consider how to support such adaptations in order to generalize the findings of the present study. Fourth, although we set the target location at 10 cm from an obstacle, the optimal target placement could differ among individuals, particularly in older adults. Previous studies have shown that the placement of the leading limb after crossing an obstacle is not only closer to the obstacle in older participants than in younger participants (27), but it also more variable (29). Such findings suggest that individual differences in older adults, influenced by factors such as walking speed and step length, contribute to variations in the optimal placement of the target for each person. This suggests that the target location is not necessarily the same for all participants. Finally, our use of a relatively short distance between the location of the obstacle and the location of the stop affected the COM velocity because participants needed to slow down after crossing. The data representing the movement patterns of leading and trailing limbs, such as foot placement of the trailing limb before an obstacle (e.g., 18.84 cm on average under the farther condition), clearance height (6.41 cm), and foot placement of the leading limb after stepping over the obstacle (40.56 cm), were generally comparable with those reported in previous studies (30, 31). Based on the findings, we concluded the movement patterns of the leading and trailing limbs, but not movement speed, are generally likely to be preserved even in our setting, which employed a short distance to the stopping point after crossing the obstacle. Further testing is necessary to ascertain whether similar results would be observed if individuals were requested to continue walking over relatively long distances after crossing the object.

In conclusion, this study demonstrated that experimentally shortening the horizontal distance between the leading limb and the obstacle can enhance collision avoidance by the trailing limb. While placing the leading limb closer to the obstacle did increase the risk of a collision involving the leading limb, this risk did not translate into a higher collision rate, likely due to more careful control. Instead, such placement appeared to improve the collision rates of the trailing limb. Future studies are necessary to examine whether such experimental manipulation could effectively improve collision avoidance behaviors, particularly in populations prone to collisions, such as older adults (9).

## Data Availability

The original contributions presented in this study are included in the article/Supplementary Material, further inquiries can be directed to the corresponding author.

## Appendix A

A total of 50 retro-reflective markers were used. Six markers were attached to the HMD, five markers to the wooden box, a single marker on each of the left and right vertical poles, and a single marker each at the start and stop positions along the walking path. On each participant's body, seven markers were attached to the trunk (the superior end of the sternum, the xiphoid process, the seventh cervical vertebra, the 10th thoracic vertebra, the right and left acromion, and the right scapula), 12 markers were attached at six locations to the left and right upper extremities (humerus, lateral epicondyle of the humerus, dorsal forearm, medial and lateral wrist joints, and dorsal third finger), four markers to the left and right superior anterior iliac spines and the left and right superior posterior iliac spines, and 12 markers were attached at six locations to the left and right lower extremities (lateral femur, lateral femoral epicondyle, lateral lower limb, external ankle joint, second metatarsal bone, and upper calcaneus bone).

## Appendix B

We conducted a preliminary study to determine the optimal foot placement of the leading limb for crossing a virtual pole in a VR environment. Participants comprised 12 young adults (29.0 ± 4.1 years old). The task was identical to that of the control condition of the main task, i.e., to walk for 3 m at a comfortable speed and step over a virtual obstacle (a long horizontal bar with a height of 20% of the lower limb length) with the right limb. We conducted five successful trials without collision and calculated the mean foot placement of the leading limb after stepping over an obstacle for each participant from the five trials. The results showed that the average value of the foot placement of the leading limb in 12 participants was 20.3 ± 7.2 cm. The mean collision rate of the trailing limb was 5 ± 9.0%, suggesting that collisions could occur under the experimental conditions. This was necessary because the purpose of our present study was to examine the effect on the collision rate of experimentally manipulating the placement of a mark to step onto. Based on the findings, we set the position of the target under the middle condition to 20 cm.
